# Structural basis of PKM2 regulation

**DOI:** 10.1007/s13238-015-0146-4

**Published:** 2015-03-17

**Authors:** Weiwei Yang

**Affiliations:** Key Laboratory of System Biology and Shanghai Key Laboratory of Molecular Andrology, Institute of Biochemistry and Cell Biology, Shanghai Institute for Biological Sciences, Chinese Academy of Sciences, Shanghai, 200031 China

Alterations in cell metabolism are a characteristic of many cancers. Cancer cells are metabolically rewired to support their rapid growth (Kim and Dang, 2006; Vander Heiden et al., 2009). The best-characterized metabolic phenotype observed in tumor cells is aerobic glycolysis, also known as the Warburg effect, which is a shift of ATP generation from high efficient oxidation phosphorylation to low efficient glycolysis even under normal oxygen concentration (Gatenby and Gillies, 2004; Warburg, 1956). Pyruvate kinase catalyzes the final step in glycolysis by transferring the phosphate from phosphoenolpyruvate (PEP) to ADP, thereby generating pyruvate and ATP (Altenberg and Greulich, 2004; Corcoran et al., 1976). In mammals, pyruvate kinase is encoded by two genes, *PKLR* and *PKM* (Noguchi et al., 1987). PKM2 is one of the splicing variants from *PKM* gene, expressed in development and most cancers, and plays a central role in tumorigenesis (Chaneton and Gottlieb, 2012; Christofk et al., 2008; Yang and Lu, 2013; Yang et al., 2012a).

The activity of PKM2 can be regulated by numerous allosteric effectors and posttranslational modifications (PTMs) that could change its conformation. For example, binding to metabolites, such as fructose-1,6-bisphosphate (FBP), can forge PKM2 into more active tetramer (Dombrauckas et al., 2005). Phosphorylation of PKM2 at tyrosine 105 inhibits the tetramer formation and pyruvate kinase activity of PKM2 (Hitosugi et al., 2009). Moreover, acetylation of residue K305 inhibits pyruvate kinase activity of PKM2 (Lv et al., 2011). Interestingly, a patient-derived mutation K422R of PKM2 (PKM2 K422R) was shown to decrease its pyruvate kinase activity in Bloom Syndrome (BS) patients (Iqbal et al., 2014). However, the detailed mechanisms underlying the regulation of PKM2 activity by those PTMs and mutations remain unclear. In this issue, Wang et al. (2015) demonstrated a structure-based mechanism for dynamic regulation of PKM2 by PTMs and a patient-derived mutation (Wang et al., 2015).

As reported in previous studies, PKM2 switches between dimer and tetramer and tetramer formation is crucial for PKM2 activation (Dombrauckas et al., 2005; Gui et al., 2013). The gel-filtration analyses of *in vitro* purified PKM2 proteins by Wang et al. showed a mixed population of PKM2 in monomer, dimer and tetramer. PKM2 WT prefers dimer under normal condition and tends to form a more active tetramer in the presence of FBP. However, acetylation-mimic mutant, PKM2 K305Q, mainly exists as a monomer, and becomes a dimer upon FBP treatment. Either monomeric or dimeric PKM2 K305Q shows much lower glycolytic activity as determined by pyruvate kinase assay. Analyzing structure, they noticed that PKM2 K305Q loses the intermolecular interactions on the A-A′ interface, which leads to the failure to form tetramer. Y105E, a phosphorylation-mimic mutation, was previously reported to inhibit PKM2 activity. In this study, it was further confirmed to prevent the FBP-induced active tetramer formation by disrupting FBP association. Taken together, these results further highlight the importance of the tetramer formation in PKM2 activation and suggest that the regulation of PKM2 oligomerization may be a general mechanism to modulate PKM2 activity.

Further investigation indicates tetrameric PKM2 does not always possess high activity. Gel-filtration analysis by Wang et al. shows that PKM2 K422R comprises of a significantly high population of tetramer. Nonetheless, much lower activity than PKM2 WT was detected with this mutant as determined by pyruvate kinase assay. This exemption makes it inaccurate to predict its activity based on PKM2 oligomerization. Interestingly, in the presence of FBP, PKM2 K422R shows a significant increase of its enzymatic activity and still maintains its tetrameric conformation. This observation implies that two types of PKM2 tetramers might exist with different activities. How do they differ from each other in structure? A recent study from Morgan et al provides a clue for further analysis (Morgan et al., 2010; Morgan et al., 2013). They showed that phenylalanine stabilizes an inactive T-state tetrameric conformer and inhibits PKM2, while FBP shifts the equilibrium to the tetrameric active R-state. This result leads to a hypothesis whether PKM2 K422R possess similar confirmation to T-state tetramer. To validate it, Wang et al. analyzed the crystal structures of PKM2 K422R in the presence or absence of FBP, and compared to R-state PKM2, PKM2^Oxalate^, or T-state PKM2, PKM2^Phe^. They found that PKM2 K422R_FBP shared R-state tetrameric conformation with PKM2^Oxalate^, whereas PKM2 K422R adopted T-state tetrameric confirmation like PKM2^Phe^. R-state PKM2 is more active than T-state PKM2. More details about these two states have been included in “Rock and Lock” model proposed by Morgan et al. However, this model does not reflect the dynamic regulation of PKM2. Wang et al. shows that PKM2 tetramer is formed by intermolecular interactions between four monomers on large (A-A′) and small (C-C′) interfaces and each individual monomer adopts similar fold with a root-mean-squared deviation of less than 0.6 Å. Further comparison of crystal structure of different PKM2 proteins indicates that PKM2 undergoes a significant confirmation change during the transition between R- and T-state. Based on these observation, they proposed a “seesaw” model to summarize conformational changes during transitions between R-/and T-state PKM2.

PKM2 R399E was previously reported to lock PKM2 in dimer and have low glycolytic activity (Gao et al., 2012). However, in crystals, PKM2 R399E adopts the T-state conformation similar to PKM2^Phe^ as shown by Wang et al. And this mutation of PKM2 inhibits the formation of the R-state conformation induced by FBP or PEP (R399E on monomer A repels E396 on monomer A and E418 on monomer B on the C-C′ interface). Consistently, PKM2 R399E is less active than PKM2 WT and less tendency to form R-state tetramer in the presence of FBP.

The important role of PKM2 in cancer makes PKM2 an attractive therapeutic target for cancer treatment. However, the complexity of PKM2 regulation enables multiple strategies to targeting PKM2. Approaches to inhibit as well as to activate PKM2 have been pursued. A chemical, TEPP-46 can enhance the intermolecular interactions on the A-A′ interface and favor an R-state conformation to activate PKM2 (Anastasiou et al., 2012). The activation of PKM2 by TEPP-46 finally rewired the metabolism of tumor cells to catabolism and inhibited the xenograft tumor growth. This result suggests a possibility to develop inhibitors or activators based on R-/T-state transition of PKM2.

Protein kinase activity of PKM2 has been recently recognized and plays critical roles in tumorigenesis (Gao et al., 2012; Yang et al., 2012b; Yang et al., 2011). This spectacular ability needs PKM2 located in nucleus. Upon EGFR activation, PKM2 was phosphorylated by ERK2, which recruits Importin α5 to facilitate its nuclear translocation (Yang et al., 2012c). In nucleus, PKM2 mainly exists as a dimer with high activity of protein kinase and low activity of pyruvate kinase. Interestingly, as shown by Wang et al. these dimers are formed with the loss of intermolecular interaction on the C-C′ interface, suggesting that regulation on this interface may be responsible for the switch from pyruvate kinase to protein kinase. Catalytic core and substrate specificity remains to be investigated.

Besides structural analysis of PKM2 regulated by PTMs, Wang et al. also examined the correlation between thermal stability and PKM2 activity. Thermal stability is the stability of a molecule at high temperatures; i.e. a molecule with more stability has more resistance to decomposition at high temperatures. The thermal stability of PKM2 was previously reported to correlate with its enzymatic activity (Morgan et al., 2010). However, Wang et al. observed an inconsistence when they performed thermal-shift assay with PKM2 WT and its PTMs-mimic mutants. They found that PKM2 R399E and PKM2 K422R are relatively more stable than PKM2 WT, suggesting PKM2 activity could not be well determined by its thermal stability either.

In conclusion, PKM2 activity is subject to complex allosteric regulation. It has been previously shown that PTMs and the association of metabolites could change PKM2 oligomerization. Tetramer formation is essential for PKM2 activation, but not all tetrameric PKM2 possess high activity. Wang et al. showed us an exemption that a patient derived mutant, PKM2 K422R is a less active tetrameric PKM2. To understand the mechanism, R- and T-state conformations of PKM2 were then recognized. Different states of PKM2 confer them distinct activities. Tetramer formation and R-/T-state transition, the regulation at these two levels provides precise control of activity of PKM2 in various physiological and pathological circumstances. Moreover, the “seesaw” model proposed not only will help us to understand the complexity of allosteric regulation, but also will provide a platform for further investigation of other modifications/mutations of PKM2, and for the development of new compounds to inhibit tumor growth based on this R-/T-state transition.
